# Safety, Pharmacokinetics, and Pharmacodynamics of Trazpiroben (TAK‐906), a Novel Selective D_2_/D_3_ Receptor Antagonist: A Phase 1 Randomized, Placebo‐Controlled Single‐ and Multiple‐Dose Escalation Study in Healthy Participants

**DOI:** 10.1002/cpdd.906

**Published:** 2021-01-18

**Authors:** Roger L. Whiting, Borje Darpo, Chunlin Chen, Margaret Fletcher, Dan Combs, Hongqi Xue, Randall R. Stoltz

**Affiliations:** ^1^ Altos Therapeutics LLC Los Altos California USA; ^2^ ERT, Inc. (previously iCardiac Technologies) Rochester New York USA; ^3^ Takeda Pharmaceuticals International Co. Cambridge Massachusetts USA; ^4^ MedAssessment, Inc. San Clemente California USA; ^5^ Combs Consulting Service Mountain View California USA; ^6^ Covance Laboratories Inc. Clinical Research Unit Evansville Indiana USA

**Keywords:** dopamine D_2_/D_3_ selective receptor antagonists, gastroparesis, pharmacodynamics, pharmacokinetics, safety

## Abstract

Gastroparesis is a chronic neuromuscular disorder of the upper gastrointestinal tract in which episodic exacerbation can lead to frequent hospitalizations and severe disability. Dopamine D_2_/D_3_ receptor antagonists have been used to treat patients with gastroparesis with some efficacy; however, their chronic use is limited owing to associated central nervous system (CNS) or cardiovascular safety concerns. Trazpiroben (TAK‐906) is a dopamine D_2_/D_3_ receptor antagonist under development for the long‐term treatment of gastroparesis. Preclinical studies in rat and dog have shown trazpiroben to have minimal brain penetration and low affinity for the human ether‐à‐go‐go‐related gene (hERG) potassium channel (IC_50_,  15.6 µM), thereby reducing the risk of the CNS and cardiovascular adverse effects seen with other dopamine D_2_/D_3_ receptor antagonists. This phase 1 trial evaluated the safety, pharmacokinetics, and pharmacodynamics of trazpiroben in healthy participants. Trazpiroben was rapidly absorbed and eliminated (T_max_, ∼1.1 hours; t_1/2_, 4–11 hours) after administration of single (5–300 mg) and multiple (50 or 100 mg) doses. Receptor target engagement was confirmed for all doses, as indicated by an increase in serum prolactin levels compared with placebo (mean prolactin C_max_, 134.3 ng/mL after administration of trazpiroben 10 mg vs 16.1 ng/mL with placebo). Therapeutically relevant single and multiple doses of trazpiroben were well tolerated in healthy participants, and no clinically meaningful cardiovascular adverse effects were observed across the whole dose range. These data support the further development of trazpiroben for the treatment of gastroparesis.

Gastroparesis is a chronic neuromuscular disorder of the upper gastrointestinal tract characterized by gastric dysrhythmia and/or delayed gastric emptying in the absence of mechanical obstruction.^1^, ^2^ The most common etiologies of gastroparesis are idiopathic, diabetic, and postsurgical, with cardinal symptoms including nausea, vomiting, abdominal pain, early satiety, and postprandial fullness.^1^, ^3^, ^4^, ^5^, ^6^, ^7^ Gastroparesis symptoms are chronic, and episodic exacerbation can lead to frequent hospitalizations and severe disability.^7^, ^8^, ^9^ Owing to challenges in diagnosis, data on global prevalence of gastroparesis are limited. A population‐based epidemiologic study from Olmsted County, Minnesota, estimated the standardized prevalence of gastroparesis to be 24.2 per 100 000 persons, and a study in the UK recently reported a standardized prevalence of 13.8 per 100 000 persons, although many individuals may remain undiagnosed.^10^, ^11^, ^12^, ^13^

Treatment options for gastroparesis include dietary control, pharmacologic therapies, and/or gastric electrical stimulation.^6^, ^14^, ^15^, ^16^ Pharmacologic therapies include antiemetics and prokinetics such as dopamine D_2_/D_3_ receptor antagonists and 5‐hydroxytryptamine 4 (5HT_4_) receptor agonists.^14^, ^15^, ^16^, ^17^

It is well established that dopamine D_2_/D_3_ receptor antagonists can reduce the symptoms of gastroparesis.^17^ Dopamine antagonists are effective in the establishment of normal gastric myoelectric activity and resolution of gastric dysrhythmias, which are reported to have a more direct relationship to the improvement of symptoms in patients with gastroparesis than gastric emptying alone.^2^, ^18^, ^19^ Furthermore, dopamine receptor antagonists have a direct antiemetic effect via inhibition of dopamine receptors in the chemoreceptor trigger zone.^20^ Metoclopramide, a combined dopamine D_2/_D_3_ receptor antagonist and 5HT_4_ receptor agonist, and domperidone, a dopamine D_2_/D_3_ receptor antagonist, are efficacious in reducing symptoms in patients with gastroparesis; however, both drugs are associated with adverse events that prohibit their chronic use.

Metoclopramide binds both peripheral and central dopamine receptors, and its chronic use is associated with extrapyramidal adverse effects. This drug is approved only for short‐term treatment and has a black box warning by the Food and Drug Administration (FDA) regarding treatment for >12 weeks.^21^, ^22^, ^23^

Domperidone does not readily cross the blood‐brain barrier and therefore does not elicit the same central nervous system (CNS) adverse effects as metoclopramide. Domperidone has been approved by the European Medicines Agency for short‐term treatment of gastroparesis, but it has not received approval from the FDA and is only available via a Single Patient Expanded Access Investigational New Drug Application owing to the risk of serious cardiac adverse effects (QT interval prolongation in electrocardiogram [ECG] recordings, likely caused by an inhibitory effect on the human ether‐à‐go‐go‐related gene [hERG] potassium channel).^24^, ^25^, ^26^, ^27^, ^28^, ^29^, ^30^, ^31^, ^32^, ^33^, ^34^, ^35^

A prokinetic therapy for gastroparesis without the potential to cause serious cardiac effects would be a valuable agent for the treatment of this disorder. Trazpiroben (previously referred to as TAK‐906 or ATC‐1906M; Figure 1) is a peripherally restricted and selective D_2_/D_3_ receptor antagonist under development for the long‐term treatment of gastroparesis. Nonclinical in vivo studies have demonstrated selective antagonism of dopamine D_2_/D_3_ receptors by trazpiroben.^36^, ^37^

**Figure 1:**
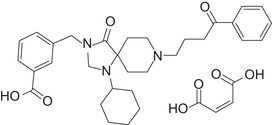
The chemical structure of trazpiroben (TAK‐906).

In safety pharmacology studies in rats, trazpiroben showed minimal effects on functional observational battery assessments and locomotion at doses exceeding those required for D_2_ receptor engagement.^38^ In further preclinical studies in rats and dogs, concentrations of trazpiroben in cerebrospinal fluid were very low compared with those in plasma after once‐daily dosing of study drug on day 4.^37^ In addition, trazpiroben did not decrease performance in rats on the accelerating rotarod, following administration of single doses associated with dopamine D_2_ receptor effects (prolactin increases).^36^ These studies indicate that trazpiroben is peripherally selective and unlikely to produce CNS effects. Trazpiroben has a low affinity for the hERG potassium channel (IC_50_, 15.6 µM). In preclinical safety pharmacology studies using telemeterized dogs, trazpiroben had no effects on QRS duration, QT_C_ duration, and ECG measurements.^38^ These studies indicate that trazpiroben has little potential to inhibit the I_kr_ potassium channel current and affect cardiac repolarization and safety.

This first‐in‐human phase 1 study in healthy participants was designed to evaluate the overall safety of trazpiroben, administered as single and multiple ascending doses, before proceeding to clinical trials in patients. This study was conducted according to the revised International Council for Harmonisation E14 guidelines (questions and answers document, December 10, 2015) to exclude clinically relevant effects of a study drug on the QT interval.

## Methods

### Study Design

In accordance with United States Title 21 Code of Federal Regulations 56, the protocol, all protocol amendments, advertisements, and informed consent forms for this study were reviewed and approved by the Midlands Independent Review Board. Written informed consent was obtained from all participants before any protocol‐specific procedures were carried out.

This phase 1 randomized, double‐blind, placebo‐controlled study was conducted in 72 healthy male and female participants (trial not registered) at Covance Clinical Research Unit, Evansville, Indiana.

The study design included a single‐ascending‐dose (SAD) study and a multiple‐ascending‐dose (MAD) study, both of which utilized an adaptive study design based on FDA Draft Guidance for Industry: Adaptive Design Clinical Trials for Drugs and Biologics. The adaptive features allowed for adjustment of trazpiroben doses by the safety committee, adjustment of sample collection times, and dose selection and washout period, based on pharmacokinetic (PK)/pharmacodynamic (PD) findings, for evaluation of the effect of food on the trazpiroben PK. For both studies, trazpiroben and placebo capsules were identical in appearance, and the Investigator and clinical research unit (CRU) staff responsible for any on‐study data collection were blinded to each participant's treatment assignment.

### SAD Study

In total, 56 participants were enrolled. After a 27‐day screening period, participants were assigned to 1 of 7 cohorts with ascending trazpiroben doses of 5, 10, 25, 50, 100, 200, or 300 mg. Each cohort comprised 8 participants, randomized to receive trazpiroben or placebo at a ratio of 6:2. The starting dose of 5 mg (0.08 mg/kg/d in a 60‐kg human) was chosen on the basis of previous toxicological studies in dogs and was well under the maximum recommended starting dose (MRSD) in humans (see Supplementary Material).

For each cohort, participants underwent an overnight fast of at least 10 hours before receiving a single oral dose of trazpiroben or placebo on day 1. Initially, 2 sentinel participants were administered trazpiroben 5 mg or placebo in a ratio of 1:1, and PK, PD, and safety data were collected for at least 48 hours. After review of these data, the remaining participants in this cohort received trazpiroben 5 mg or placebo at a ratio of 5:1. Subsequent cohorts at escalating doses were staggered by 48 hours to allow a review of all available PD data, precluding the need for sentinel dosing. Safety observations (vital signs and safety ECGs) were carried out from 1‐hour predose through to 24 hours postdose. Participants were discharged from the CRU on day 2 and returned for final safety assessments 5–8 days after administration of study drug.

#### Effect of Food

Participants in the trazpiroben 25‐mg cohort received a second 25‐mg dose in the fed state to evaluate the effect of food on the PK of trazpiroben. On day 3, after an overnight fast of at least 10 hours, participants received a standardized high‐fat, high‐calorie meal for consumption within 20 minutes. Trazpiroben or placebo was administered 30 minutes after the start of the meal.

Participants were observed from administration of the first dose (in the fasted state) through 36 hours after the second dose (in the fed state). Participants were discharged from the CRU on day 4 and returned for final safety assessments 5–8 days after the second administration of study drug.

### MAD Study

The MAD study was initiated on completion of all cohorts and evaluation of all participants in the SAD study.

After a 27‐day screening period, 16 participants were enrolled across 2 dose cohorts: trazpiroben 50 mg (n  =  8) and trazpiroben 100 mg (n  =  8). In each cohort, participants were randomized to receive trazpiroben or placebo at a ratio of 6:2. The starting dose of trazpiroben (50 mg twice daily) was determined by the safety committee based on data from the SAD study.

After an overnight fast of least 10 hours, participants received trazpiroben (50 or 100 mg) or placebo orally twice daily on days 1–4 and a single dose on day 5. Administration of the study drug in the 100‐mg cohort was initiated after a review of 48 hours of PK and safety data from the 50‐mg cohort. On days 1–4, the evening dose was administered 12 hours after the morning dose and ∼3 hours after commencing a meal. An evening dose was not administered on day 5. Participants were discharged from the CRU on day 6 and returned to the CRU for final safety assessments 5–8 days after discharge.

### Inclusion and Exclusion Criteria

Healthy male and female participants were aged from 18 to 60 years with a body mass index (BMI) ranging from 18 to 32 kg/m^2^. Participants with a history of hyperprolactinemia, pituitary adenoma, and/or hyperthyroidism or an abnormal electrocardiographic finding (baseline QT interval corrected for heart rate using Fridericia's correction [QTcF] > 430 milliseconds) were excluded.

### Study Assessments

#### Pharmacokinetic Analysis

In the SAD study, blood samples were collected 1‐hour predose and 0.25, 0.5, 1, 1.5, 2, 3, 4, 6, 8, 12, 16, and 24 hours postdose and 36 hours postdose for the 5‐, 10‐, and 25‐mg cohorts. In the MAD study, blood samples were collected after the morning dose, serially on day 1 (1 hour predose to 12 hours after first dose) and day 5 (1 hour predose to 24 hours postdose) and on intermittent days in‐between (days 2, 3, and 4 predose and 1, 2, and 4 hours postdose). Analysis of trazpiroben concentrations in plasma was determined using a validated analytical method employing liquid chromatography and mass spectroscopy (see Supplementary Material). PK parameters for trazpiroben were determined using noncompartmental techniques according to standard methods and WinNonlin version 6.1 software (Certara Corp., Princeton, New Jersey).^39^ Drug PK parameters measured included time to reach maximum plasma concentration (T_max_), apparent terminal elimination half‐life (t_1/2_), maximum plasma drug concentration (C_max_), area under the plasma concentration‐versus‐time curve (AUC), and apparent oral clearance (CL/F).

#### Pharmacodynamic Analysis

Serum prolactin concentrations were measured as a biomarker for dopamine D_2_ receptor antagonism. In the SAD study, blood samples for serum prolactin measurements were collected predose and 1, 1.5, 2, 3, 4, 6, 12, 24, and 36 hours postdose. In the MAD study, blood samples were collected predose and serially on days 1 and 5. Analysis of serum prolactin concentration was performed using the ADVIA Centaur Commercial Assay (see Supplementary Material). The determined serum prolactin parameters included C_max_, T_max_, AUC, and t_1/2_.

#### Cardiodynamic Assessment

Continuous 12‐lead ECG (Holter) recording was performed from 1 hour predose through to 24 hours postdose in the SAD study. In the MAD study, continuous 12‐lead ECG (Holter) recording was performed on day 1 from 1 hour predose through 12 hours after the morning dose and on day 5 from 1 hour predose through 24 hours after the morning dose. ECG data for concentration‐QT analysis were collected using a Global Instrumentation (Manlius, New York) MI2R ECG continuous 12‐lead digital recorder. Using the Expert Precision QT technique, 12‐lead ECGs were extracted by the central ECG laboratory (ERT, Rochester, New York) in up to 10 replicates at times paired with PK samples on day 1 in the SAD and MAD parts of the study and on day 5 in the MAD study.

#### Safety and Tolerability

Measurement of vital signs and standard 12‐lead safety ECG assessments were performed predose and 0.25, 0.5, 1, 1.5, 2, 3, 4, 6, 8, 12, 16, 24, and 36 hours postdose. Clinical laboratory tests (hematology, clinical chemistry, and urinalysis) and physical examinations were performed predose and 8, 24, and 36 hours postdose. Telemetric ECG monitoring was conducted in place of standard 12‐lead ECG assessments for 12 hours from day −1 until predose on day 1, after which cardiac activity was evaluated via Holter monitor. All physical examinations included a neurological examination and, as part of the adaptive study design, additional CNS examinations (including cognitive testing) could be performed, according to the investigator's clinical judgment and if sedation was reported. Adverse events were monitored continuously throughout the SAD and MAD parts of the study.

### Statistical Methods

The sample size chosen for both the SAD and MAD parts of the study was based on first‐in‐human SAD and MAD studies of a similar nature and was not based on power calculations. PK and PD data analyses were performed using statistical software SAS version 9.3, WinNonlin version 6.1, and Microsoft Excel 2010. All PK and PD parameters were summarized by cohort and study day and compared with pooled data from the placebo cohorts.

The PK analysis set included all participants who received at least 1 dose of study drug and for whom at least 1 postdose sample result for trazpiroben was reported. The PD analysis set included all participants who received at least 1 dose of study drug and for whom at least 1 postdose serum prolactin result was reported. Baseline for the PD analysis was the predose concentration on day 1 (SAD and MAD parts of the study) and day 5 (MAD study only). The statistical methodology for the analysis of dose‐response for PK and PD parameters, trazpiroben accumulation, time dependence of trazpiroben kinetics, and effect of food on the bioavailability of trazpiroben are provided in the Supplementary Material.

Data analyses for cardiodynamic assessment were performed using statistical software SAS version 9.4. The QT/QTc analysis set included all participants who received at least 1 dose of study drug for whom measurements were available at baseline as well as on‐study and for whom ECG data were available for at least 1 point with a valid change‐from‐baseline QTcF (ΔQTcF) value. For participants in the SAD study 25‐mg cohort, data from both day 1 (fasted) and day 3 (fed) were included. The PK/QTc analysis set included all participants in the QT/QTc analysis set with at least 1 pair of postdose PK and QTc data from the same point as well as participants in the QT/QTc analysis set who received placebo. Baseline for cardiodynamic assessments was defined as the average of the measured ECG intervals from 3 predose times taken within 1 hour on day 1.

The plasma trazpiroben concentration‐QTc analysis was the primary cardiodynamic analysis. The relationship between ∆QTcF and plasma trazpiroben concentrations for doses of 5, 10, and 25 mg fasted (day 1), 25 mg fed (day 3), 50, 100, 200, and 300 mg was investigated by a linear mixed‐effects modeling approach. The model used ΔQTcF as the dependent variable, plasma trazpiroben concentration as the covariate, treatment (active or placebo), and time as fixed effects, and a random intercept per participant. In all calculations, 0 was substituted for concentrations below the quantification limit of the assay and for concentrations in participants receiving placebo. The degrees of freedom for the model estimates were determined by the Kenward‐Rogers method. From the model, the slope (ie, the regression parameter for the concentration) and the treatment effect‐specific intercept (defined as the difference between active and placebo) were estimated together with 2‐sided 90% confidence intervals (CIs). The estimates for the time effect were reported with degrees of freedom and standard errors.

The geometric mean of the individual C_max_ values for participants in each trazpiroben dose cohort was determined. The predicted effect and its 2‐sided 90%CI for the placebo‐corrected ΔQTcF (ie, slope estimate × concentration + treatment effect‐specific intercept) at this geometric mean C_max_ were obtained separately for each trazpiroben dose cohort.

Exploratory analyses (via graphical displays and/or model fitting) included accounting for a delayed effect and the justification for the choice of pharmacodynamic model (linear vs nonlinear). Safety data were listed and summarized using descriptive and summary statistics for participants included in the safety set (all participants who received at least 1 dose of study drug).

## Results

### Study Disposition

In the SAD study, 56 participants were enrolled in 7 dose cohorts of 8 participants each, and 55 participants completed the study. One participant from the 200‐mg cohort was lost to follow‐up. All assessments were completed for this participant except for the scheduled follow‐up safety assessments.

In the MAD study, 16 participants were enrolled in 2 dose cohorts each of 8 participants, of whom 15 participants completed the study. One participant in the 100‐mg twice‐daily cohort discontinued owing to a family emergency and withdrew consent on day 3, after receiving the first 4 doses of trazpiroben 100 mg.

### Participant Demographics

In the SAD study, 32 male and 24 female participants were enrolled across all dose cohorts, with a mean ± standard deviation (SD) age of 38 ± 10.1 years, body weight of 79.8 ± 13.8 kg, and BMI of 26.9 ± 3.2 kg/m^2^. Participants were predominantly white (51.8%) or black African American (42.9%).

In the MAD study, 10 male and 6 female participants were enrolled across both dose cohorts, with a mean ± SD age of 30 ± 10.6 years, body weight of 79.6 ± 16.0 kg, and BMI of 26.7 ± 3.8 kg/m^2^. Participants were predominantly white (50.0%) or black African American (37.5%).

No meaningful differences were observed across cohorts within each study or between the SAD and MAD study populations.

### Pharmacokinetics in SAD Study

**Figure 2:**
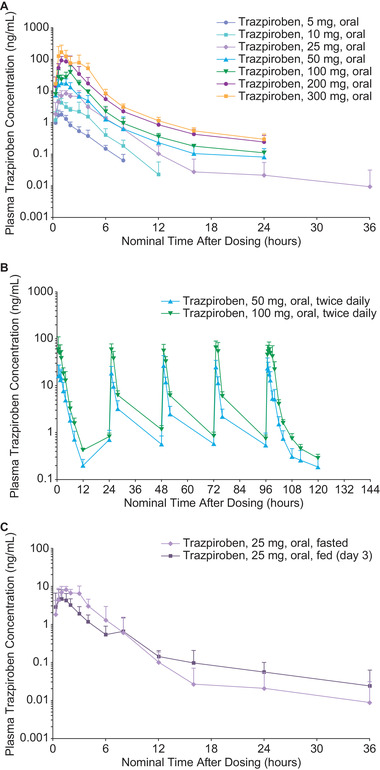
Plasma trazpiroben concentration (mean ± standard deviation; semilog scale) versus nominal time in fasting participants for (A) the single‐ascending‐dose study, (B) the multiple‐ascending‐dose study, and (C) in fasted versus fed participants on day 3 of the single‐ascending‐dose study. All values below the level of quantification were taken as 0 for the calculation of the mean.

A summary of the key PK parameters from the SAD study is provided in Table 1. Trazpiroben was rapidly absorbed and eliminated in all dose cohorts (Figure 2A). Median plasma trazpiroben T_max_ was 1.08 hours (range, 0.3–3.0 hours), and mean plasma trazpiroben t_1/2_ was ∼4.0 hours (range, 0.8–23.2 hours). For single oral doses of 5 and 10 mg, the mean plasma trazpiroben t_1/2_ was ∼1.6 hours, and at doses of 25–300 mg, the mean plasma trazpiroben t_1/2_ ranged from 3.1 to 6.0 hours in fasted participants; the difference may be attributable to the large number of samples from the 5‐ and 10‐mg cohorts being below the lower limit of quantification (LLOQ) for trazpiroben (LLOQ, 0.050 ng/mL).

**Table 1:** Summary of Trazpiroben PK Parameters for the Single‐Ascending‐Dose Study and the Multiple‐Ascending‐Dose Study

Trazpiroben Dose, mg	Day (Fed/Fasted State)		T_max_, h	C_max_, ng/mL	AUC_last_, h · ng/mL	AUC_∞_, h · ng/mL	AUC_0‐12_, h · ng/mL	CL/F, L/h	t_1/2_, h
Single‐ascending‐dose study									
5	1 (fasted)	n	6	6	6	6	6	6	6
		Mean	0.8	2.1	4.3	4.5	4.5	970	1.6
		SD	0.3	0.5	1.2	1.2	1.2	285.6	0.3
10	1 (fasted)	n	6	6	6	6	6	6	6
		Mean	1.2	6.2	12.9	13.1	13.0	669	1.6
		SD	0.9	1.9	4.5	4.4	4.4	166.8	0.2
25	1 (fasted)	n	6	6	6	5	6	5	5
		Mean	1.8	10.5	30.5	30.4	30.0	710	6.0
		SD	1.0	0.7	7.6	9.0	6.7	157.6	9.6
	3 (fed)	n	6	6	6	4	6	4	4
		Mean	2.2	6.7	17.6	17.2	16.2	1191	7.0
		SD	2.9	2.9	4.9	1.2	3.8	79.8	6.0
50	1 (fasted)	n	6	6	6	4	6	4	4
		Mean	1.0	22.0	53.2	56.7	51.9	742	3.1
		SD	0.5	6.9	12.0	11.6	12.1	141.2	0.4
100	1 (fasted)	n	6	6	6	5	6	5	5
		Mean	1.8	48.3	103.8	108.2	101.6	791	5.4
		SD	0.8	23.1	23.7	24.7	23.7	196.9	1.4
200	1 (fasted)	n	6	6	6	6	6	6	6
		Mean	1.2	105.6	240.1	242.4	234.9	779	5.43
		SD	0.5	43.5	111.9	111.4	111.4	303.9	3.5
300	1 (fasted)	n	6	6	6	5	6	5	5
		Mean	1.9	191.8	421.3	446.5	414.6	584	5.1
		SD	0.9	108.9	126.0	124.9	127.3	159.0	1.3
Multiple‐ascending‐dose study									
50	1 (fasted)	n	6	6	6	6	6	6	6
		Mean	1.6	23.3	53.6	54.1	53.6	837	2.0
		SD	0.8	8.0	18.0	18.0	18.0	313.3	0.5
	5 (fasted)	n	6	6	6	4	6	4	4
		Mean	1.2	31.2	60.9	68.3	58.1	650	11.0
		SD	0.5	9.6	14.7	11.7	13.9	93.1	1.2
100	1 (fasted)	n	6	6	6	6	6	6	6
		Mean	1.0	71.5	152.6	153.8	152.6	594	2.1
		SD	0.6	47.0	62.5	62.5	62.5	198.0	0.4
	5 (fasted)	n	5	5	5	4	5	4	4
		Mean	1.5	71.6	205.9	224.7	200.5	404	6.2
		SD	0.5	20.9	72.9	72.0	72.8	108.4	2.7

AUC_last_, area under the concentration‐time curve (AUC) from 0 to T_last_; AUC_0‐12_, AUC over the dosing interval from time 0 to 12 hours; AUC_∞_, AUC from time 0 to infinity; C_max_, maximum peak observed concentration within the dosing interval; CL/F, apparent drug clearance; mean, arithmetic mean; n, number of observations; SD, standard deviation; t_1/2_, apparent elimination half‐life; T_max_, time to maximum concentration within the dosing interval; Vz/F, apparent volume of distribution.

The arithmetic mean plasma trazpiroben C_max_ ranged from 2.1 ng/mL in the 5‐mg dose cohort to 191.8 ng/mL in the 300‐mg dose cohort. The highest individual value for trazpiroben C_max_ was 398 ng/mL, which was observed for the 300‐mg dose. The mean ratio of exposure ratio to dose ratio for all PK parameters evaluated under fasted conditions ranged from 1.16 to 1.18 across all doses and from 1.09 to 1.10 when adjacent doses were compared, indicating only minor deviation from dose proportionality. Across the 25‐ to 300‐mg dose cohorts, mean CL/F of trazpiroben ranged from ∼600 to ∼800 L/h. The large number of samples below the lower limit of quantification for the 5‐ and 10‐mg doses precluded meaningful calculation of this parameter at the lower doses.

#### Effect of Food

On the basis of a demonstrated pharmacological effect of trazpiroben in the 5‐ and 10‐mg cohorts, a single 25‐mg dose of trazpiroben was chosen to assess effect of food on the PK of trazpiroben because the plasma concentration of trazpiroben had been shown to be above the lower limit of detection in all samples for the 25‐mg dose. In comparison with the fasted state, food reduced absorption of trazpiroben 25 mg in the same cohort (Figure 2C). The fed‐state‐to‐fasted‐state ratio of mean plasma trazpiroben C_max_ was 58.1% (90%CI, 39.0–86.5%), the ratio of area under the concentration‐versus‐time curve from time zero to infinity (AUC_inf_) was 58.2% (90%CI, 45.1–75.2%), and the ratio of AUC from time zero to 12 hours postdose (AUC_0‐12_) was 53.7% (90%CI, 42.8–67.4%). Confidence interval testing showed that all parameters were generally outside the comparison interval of 80–125%. There was no effect of food on trazpiroben T_max_.

### Pharmacokinetics in MAD Study

A summary of the key PK parameters from the MAD study is provided in Table 1. Rapid absorption and elimination of trazpiroben was also observed in the MAD study (Figure 2B). On day 1, median plasma trazpiroben T_max_ was 1.3 hours (range, 0.7–3.0 hours) and 0.7 hours (range, 0.7–2.0 hours), and mean ± SD plasma trazpiroben t_1/2_ was 2.0 ± 0.5 and 2.1 ± 0.4 hours for doses of 50 and 100 mg twice daily, respectively (calculated from plasma samples collected over 12 hours). On day 5, median plasma trazpiroben T_max_ was 1.1 hours (range, 0.7–2.0 hours) and 1.5 hours (range, 0.7–2.0 hours), and mean ± SD trazpiroben *t*_1/2_ was 11.0 ± 1.2 and 6.2 ± 2.8 hours for 50‐ and 100‐mg twice‐daily doses, respectively (calculated from plasma samples collected over 24 hours).

After administration of trazpiroben 50 mg twice daily, mean plasma C_max_ was 23.3 ± 8.0 and 31.2 ± 9.6 ng/mL on day 1 and day 5, respectively. For the dose of 100 mg twice daily, C_max_ was 71.5 ± 47.0 and 71.6 ± 20.9 ng/mL on day 1 and day 5, respectively. The highest individual C_max_ was 165 ng/mL in a participant receiving trazpiroben 100 mg twice daily.

On day 1, there was no effect of trazpiroben dose on the nonexposure parameter CL/F. On day 5, mean values for trazpiroben CL/F were ∼650 and ∼404 L/hour for the 50‐ and 100‐mg twice‐daily doses, respectively.

On day 5, the mean C_avg_ was 4.8 and 16.7 ng/mL, the mean C_min_ was 0.3 and 0.7 ng/mL, and the mean accumulation index (the predicted accumulation from single dose to steady state) was 1.9 and 1.4 for the 50‐ and 100‐mg twice‐daily doses, respectively.

### Pharmacodynamics in SAD Study

**Figure 3:**
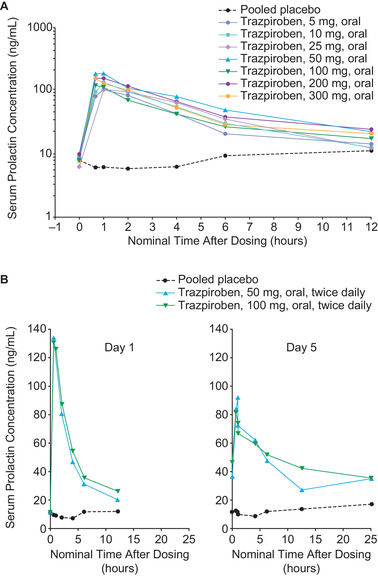
Serum prolactin concentration versus nominal time in fasting participants in (A) the single‐ascending‐dose study and (B) on day 1 and day 5 in the multiple‐ascending‐dose study (mean; standard deviations for mean serum prolactin C_max_ are provided in Table 2).

Mean serum prolactin C_max_ increased from 16.1 ng/mL in the pooled placebo cohorts to 98.2–188.9 ng/mL across all trazpiroben doses (Figure 3A, Table 2). The increases in serum prolactin levels were rapid (median serum prolactin T_max_, 1.1 hours; range, 0.7–2.0 hours across all 48 single‐dose trazpiroben administrations) and transient (mean serum prolactin t_1/2_, 10.8 hours; range, 4.8–21.0 hours across the 12 trazpiroben administrations for which t_1/2_ could be calculated). There was a minimal difference in prolactin response between the 10‐ and 300‐mg doses, as indicated by small differences in the ratio of prolactin levels relative to placebo in these dose cohorts: serum prolactin C_max_ ratios of 8.3 and 9.2 and serum prolactin AUC_0‐12_ ratios of 5.1 and 5.6 in the 10‐ and 300‐mg cohorts, respectively. In comparison, the C_max_ ratio was 6.1, and serum prolactin AUC_0‐12_ ratio was 4.2 in the trazpiroben 5‐mg cohort.

**Table 2:** Serum Prolactin Dose‐Response in Fasting Participants in the Single‐Ascending‐Dose Study

Trazpiroben Dose, mg^a^	Prolactin C_max_ (ng/mL), Mean (SD)	Ratio of Trazpiroben Treated:Pooled Placebo C_max_	Prolactin AUC _0‐12_ (h · ng/mL), Mean (SD)	Ratio of Trazpiroben Treated:Pooled Placebo AUC_0‐12_
5	98.2 (69.6)	6.1	433.4 (264.4)	4.2
10	134.3 (85.0)	8.3	533.6 (324.4)	5.1
25	129.0 (86.2)	8.0	543.2 (231.5)	5.2
50	188.9 (171.6)	11.7	776.6 (673.5)	7.5
100	118.1 (66.3)	7.3	474.2 (194.3)	4.6
200	175.6 (93.5)	10.9	700.4 (356.9)	6.7
300	147.7 (98.1)	9.2	584.1 (475.4)	5.6
Pooled placebo (n = 14)	16.1 (7.4)	—	104.2 (49.8)	—

C_max_, maximum peak observed concentration within the dosing interval; AUC_0‐12_, area under the concentration‐time curve over the dosing interval from time 0 to 12 hours.

^a^ Six participants per trazpiroben dose group.

### Pharmacodynamics in MAD Study

Increases in serum prolactin concentration were also rapid in the MAD study: median serum prolactin T_max_ was 0.7 hours (range, 0.7–1.1 hours) for both doses on day 1 and day 5. Mean serum prolactin t_1/2_ in participants who received multiple doses of trazpiroben 50 or 100 mg was 13.5 hours (range, 4.2–62.3 hours). Serum prolactin C_max_ was similar for both doses and was several‐fold higher than in the pooled placebo cohorts (Figure 3B).

### Cardiodynamic Assessment

**Figure 4:**
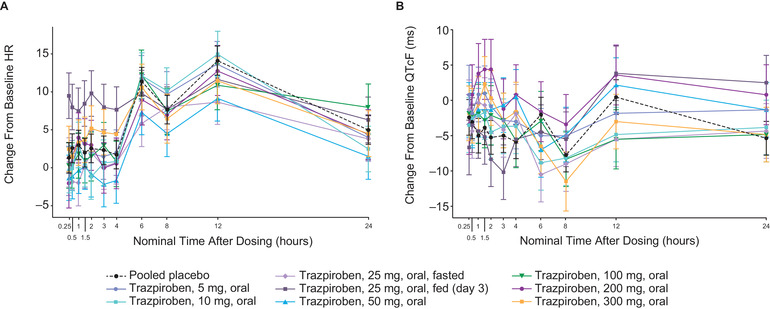
Change from baseline in (A) heart rate and (B) QTcF across times in the single‐ascending‐dose study. HR, heart rate; QTcF, QT interval corrected for heart rate using Fridericia's correction.

Mean change from baseline heart rate (ΔHR) was small in all fasted cohorts, whereas the effect of food could be observed, with a larger ΔHR in fed participants (Figure 4A). A single oral dose of trazpiroben (5–300 mg) did not have a relevant effect on cardiac conduction (PR and QRS intervals). ∆QTcF was very small or negative across postdose times in all dose cohorts (Figure 4B). The largest QT effect was observed in the trazpiroben 200‐mg cohort, in which mean ΔQTcF peaked at 4.4 milliseconds at 1.5 and 2 hours postdose. In the highest dose cohort, trazpiroben 300 mg, the mean ΔQTcF peaked at 2.3 milliseconds at 1.5 hours postdose.

Placebo‐corrected ΔHR and ΔQTcF are shown in Table S1. At 1.0, 1.5, and 2.0 hours in the 200‐mg cohort, the mean placebo‐corrected ΔQTcF reached 8.8, 8.3, and 9.6 milliseconds, respectively, but did not further increase in the 300‐mg cohort, in which the mean placebo‐corrected ΔQTcF was 4.5, 6.2, and 5.6 milliseconds, respectively. The QTcF did not exceed 450 milliseconds in any participants at any postdose time.

**Figure 5:**
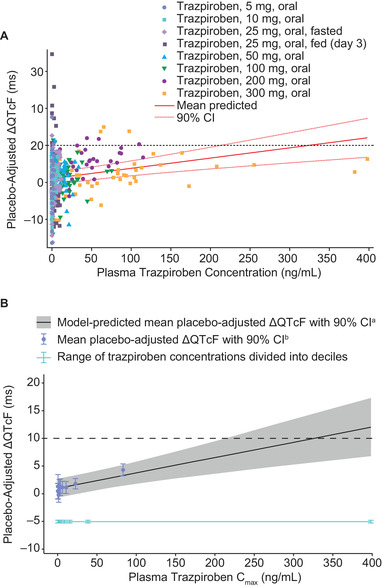
(A) Relationship between plasma trazpiroben concentration and placebo‐corrected ΔQTcF, and (B) model‐predicted placebo‐corrected ΔQTcF and estimated placebo‐adjusted ΔQTcF (mean and 90%CI) across deciles of plasma trazpiroben concentrations in the single‐ascending‐dose study. In B, the solid black line with gray‐shaded area denotes the model‐predicted mean placebo‐corrected ΔQTcF (ΔΔQTcF) with 90%CI, which is calculated from the equation ΔΔQTcF = 1.0 + 0.03 × trazpiroben. The filled circles with vertical bars denote the estimated mean placebo‐adjusted ΔQTcF with 90%CI displayed at the associated median plasma concentration within each decile for trazpiroben, among which the individually estimated placebo‐adjusted ΔQTcF*_i,k_* (ΔΔQTcF*_i,k_*) equals the individual ΔQTcF*_i,k_* for participant *i* administered with trazpiroben at time *k* minus the estimation of time effect at time *k*. The horizontal line with notches shows the range of concentrations divided into deciles for trazpiroben. The area between each decile represents the point at which 10% of the data are present; the first notch to second notch denotes the first 10% of the data, the second notch to third notch denotes the next 10% of the data, and so forth. QTcF, QT interval corrected for heart rate using Fridericia's correction.

A linear mixed‐effects model with a treatment effect‐specific intercept provided an acceptable fit to the observed QTcF and PK data and was therefore used to establish the relationship between plasma trazpiroben concentration and ΔQTcF. The slope of concentration against ΔQTcF was slightly positive and statistically significant (0.0277 milliseconds per ng/mL; 90%CI, 0.0146–0.0407 milliseconds per ng/mL; Figure 5A). Using this model, the predicted QT effect (placebo‐corrected ΔQTcF) at the observed geometric mean peak plasma concentration after the 2 highest doses of trazpiroben (200 mg: geometric mean plasma trazpiroben C_max_, 87 ng/mL; 300 mg: geometric mean plasma trazpiroben C_max_, 171 ng/mL) was 3.5 milliseconds (90%CI, 1.5–5.4 milliseconds) and 5.8 milliseconds (90%CI, 3.1–8.4 milliseconds), respectively (Figure 5B). An effect on placebo‐corrected ΔQTcF exceeding 10 milliseconds could be excluded for plasma trazpiroben concentrations below ∼200 ng/mL.

### Safety and Tolerability

No serious adverse events (AEs) or severe AEs were reported during the study, and no participants discontinued owing to an AE. Sedation was not reported or observed in any participant; therefore, additional CNS examinations and cognitive tests were not required in the SAD or MAD study.

In the SAD study, 7 participants (12.5%) experienced a total of 8 treatment‐emergent AEs (TEAEs). No TEAE was reported by >1 participant receiving trazpiroben. All TEAEs were mild in intensity other than 1 episode of moderate severity bronchitis in a participant from the 5‐mg cohort receiving the lowest dose of trazpiroben (5 mg). Two participants experienced a TEAE considered possibly related to the study drug (breath odor or abdominal pain). The only nervous system disorder reported was headache, reported by 1 of 14 participants (7.1%) receiving placebo and 1 of 6 participants (16.7%) receiving trazpiroben 50 mg. There was no trend for increased incidence or severity of TEAEs with increasing doses of trazpiroben, and the incidence of TEAEs for the pooled placebo participants was similar to that in participants receiving trazpiroben.

In the MAD study, 7 participants (43.8%) experienced a total of 9 TEAEs. Incidence of TEAEs was similar in participants receiving trazpiroben 50 mg twice daily, 100 mg twice daily, and placebo. All TEAEs were mild in intensity. Nervous system disorders reported were headache (trazpiroben 50 and 100 mg twice daily, 16.7% of participants for each dose cohort), dizziness (placebo: 25%; trazpiroben 100 mg twice daily: 33%) and presyncope (trazpiroben 50 mg twice daily: 16.7%). One participant in the 100‐mg cohort experienced 2 TEAEs considered possibly related to study drug (fatigue and dizziness). Only single episodes of dizziness or presyncope were reported despite repeat dosing of trazpiroben.

There were no apparent treatment‐related trends in mean clinical chemistry, hematology, or urinalysis measurements, vital sign values, or mean 12‐lead ECG parameters in either study. No abnormal physical examination findings were noted during the study, and there were no notable changes in body weight.

## Discussion

Dopamine D_2_/D_3_ receptor antagonists can reduce the symptoms of gastroparesis via a number of mechanisms. However, the chronic use of currently available dopamine receptor antagonists, the selective D_2_/D_3_ antagonist domperidone and the D_2_/D_3_ antagonist and 5HT_4_ agonist metoclopramide, is limited owing to the risk of AEs. Trazpiroben (TAK‐906) is a potent, peripherally restricted, and selective dopamine D_2_/D_3_ receptor antagonist that offers the potential to avoid the CNS and cardiovascular AEs reported for metoclopramide and domperidone, respectively.

Our study evaluated a range of trazpiroben doses from 5 to 300 mg. The starting dose was based on observations in preclinical pharmacodynamic studies that measured prolactin increases in rats and inhibition of apomorphine‐induced emesis in dogs, and in a toxicology study in dogs, the most sensitive species (see Supplementary Material).^40^ Pharmacodynamic effects were observed in both species at ∼0.1 mg/kg, with maximal effects at 1 mg/kg. Mild sedation, the first clinical sign in humans of CNS‐mediated effects of dopamine D_2_ antagonists, was observed in dogs at 10 and 50 mg/kg/d. From the dog toxicology study, the MRSD in humans was determined as 168 mg (2.8 mg/kg/d for a 60‐kg human). On the basis of the peripherally mediated pharmacodynamic effects observed at low doses in the preclinical studies and to ensure that sedation was avoided in humans, we opted to initiate dosing at 5 mg (0.08 mg/kg/d).

Pharmacokinetic analysis showed that trazpiroben was rapidly absorbed and eliminated. Peak plasma trazpiroben concentrations were reached after ∼1.1 hours, after both single and multiple doses of trazpiroben and for all cohorts. The terminal elimination half‐life of trazpiroben was ∼4 hours after all single doses and 11 and 6.2 hours after twice‐daily doses of 50 and 100 mg, respectively. In the SAD study, mean plasma trazpiroben t_1/2_ values in the 5‐ and 10‐mg cohorts were shorter (t_1/2_, ∼1.6 hours) than in the 25‐ to 300‐mg cohorts (t_1/2_, 3.1–6.0 hours). This may reflect the large number of samples from 5‐ and 10‐mg cohorts that were below the lower limit of quantitation for trazpiroben. In the MAD study, trazpiroben t_1/2_ values calculated for day 1 were lower than for day 5, although calculations may have been influenced by the plasma samples only being collected to 12 hours on day 1 compared with 24 hours on day 5.

Exposure of trazpiroben was dose dependent, and food reduced exposure to trazpiroben by ∼40%, compared with fasted participants, after a dose of 25 mg. Although the mechanism for this food effect is unknown, it is usual for patients with gastroparesis to take their medication before food (eg, 1 hour prior to meals) to prevent the onset of symptoms and hence the reduced exposure of trazpiroben with food is likely to be circumvented. There was no effect of dose on the nonexposure parameters CL/F, t_1/2_, and MRT_inf_. Accumulation of trazpiroben between twice‐daily doses, 12 hours apart, was minor (C_max_ and AUC_0‐12_ ratios for day 5 to day 1 were <40% and <30%, respectively).

Increases in serum prolactin concentration were used as a biomarker for target engagement of trazpiroben.^41^ A substantial and rapid increase in serum prolactin concentration was observed for all doses of trazpiroben but not for placebo. There was a trend toward a plateaued increase in prolactin at a dose of 10 mg trazpiroben, indicating maximal target inhibition by trazpiroben had been reached at this dose. Increases in serum prolactin concentration were transient after single‐dose administration of trazpiroben, and serum prolactin accumulation was negligible with twice‐daily dosing over 5 days. Because approved dopamine D_2_ receptor antagonists (such as metoclopramide) have labels that include AEs associated with hyperprolactinemia (eg, impaired reproductive function), longer‐term administration during the planned phase 2 and 3 studies will be used to monitor for potential adverse events associated with elevated prolactin levels.

Given that current dopamine D_2_/D_3_ antagonists used for treatment of gastroparesis are associated with CNS and cardiovascular adverse effects, the effects of trazpiroben on these safety outcomes were of interest. Preclinical studies in rat and dog have shown minimal brain penetration of trazpiroben, and safety pharmacology studies have shown negligible locomotor effects of trazpiroben in rats.^37^, ^38^ As cited above, class‐related pharmacological effects (including signs of mild sedation) in dogs have been noted; however, no clinical signs of sedation were observed in any participants in this study, providing further evidence that trazpiroben has minimal brain penetration at the doses administered in this study.

Unlike domperidone, the affinity of trazpiroben for the hERG potassium channel is weak (IC_50_, 15.6 µM for trazpiroben, compared with 57 nM for domperidone), and no ECG abnormalities attributable to administration of trazpiroben have been reported in safety pharmacology studies (see Supplementary Material).^42^ No cardiovascular AEs were observed in either the SAD or MAD parts of this study, as demonstrated by the absence of clinically meaningful changes in QT in the ECG assessments.

Using cardiodynamic analysis of data from the SAD part of the study, plasma trazpiroben concentrations of up to 200 ng/mL are predicted to have no clinically meaningful QT effects (ie, placebo‐corrected ΔQTcF not greater than 10 milliseconds). The maximal pharmacodynamic response (indicated by a plateau in serum prolactin increases) was reached at trazpiroben 10 mg, for which the mean plasma trazpiroben concentration was 6.2 ng/mL, indicating a substantial margin between an effective dose and the dose predicted to have no cardiovascular adverse effects. On the basis of the preliminary PK/PD results from the SAD study, the cardiodynamic evaluation and C‐QTc analysis were not performed in the MAD study (although cardiodynamic measurements and safety ECG assessments were performed and showed no clinically meaningful cardiac adverse effects).

No serious AEs or severe AEs were reported during the study, and no participant discontinued owing to an AE. The overall incidence of TEAEs was low, and there was no relationship between incidence or severity of TEAEs and trazpiroben dose. The incidence of TEAEs in the participants receiving placebo or trazpiroben was similar. Further studies are planned to evaluate the drug exposure margin in patients with impaired clearance because of hepatic or renal impairment. Further investigation of the safety and tolerability of trazpiroben in patients with gastroparesis has been conducted in a phase 2a study (NCT03268941, completed), and a phase 2b study with dosing over 3 months is in progress (NCT03544229).

## Conclusions

Therapeutically relevant single and multiple doses of trazpiroben were rapidly absorbed and eliminated and well tolerated in healthy participants. Receptor target engagement by trazpiroben was observed at all doses administered, and maximal target engagement was reached at trazpiroben plasma concentrations significantly below the highest concentrations expected to show no clinically meaningful cardiovascular adverse effects. Trazpiroben has the potential to fulfill the unmet clinical need for an efficacious gastroparesis treatment with a safety profile compatible with long‐term use.

## Conflicts of Interest

R.L.W., PhD, a former shareholder of Altos Therapeutics LLC, will benefit from any future payments by Takeda with respect to certain clinical development and commercial milestones for trazpiroben. R.L.W. has received consulting fees from Takeda. B.D., MD, PhD, is a consultant for ERT, owns stock, and is eligible for stock options. C.C., MD, PhD, was an employee of Takeda Pharmaceuticals International Co. at the time of this study and was eligible for stock/stock options. M.F., MD, PhD, is the owner of MedAssessment, Inc. and was a consultant for Altos Therapeutics LLC at the time of this study. D.C., BS, is the owner of Combs Consulting Service. H.X., PhD, is an employee of ERT Inc. R.R.S., MD, was an employee of Covance Laboratories Inc. at the time of the study.

## Funding

This study was sponsored by Altos Therapeutics LLC. Medical writing support was provided by Sally McTaggart, PhD. of Oxford PharmaGenesis, Oxford, UK, and was funded by Takeda Pharmaceutical Company Ltd.

## Data‐Sharing Statement

The data sets, including the redacted study protocol, redacted statistical analysis plan, and individual participants data supporting the results reported in this article, will be made available within 3 months from initial request to researchers who provide a methodologically sound proposal. The data will be provided after its deidentification, in compliance with applicable privacy laws, data protection, and requirements for consent and anonymization.

## Supporting information

Supporting Information.
