# Supplementary material for: Safety, Pharmacokinetics, and Pharmacodynamics of Trazpiroben (TAK‐906), a Novel Selective D2/D3 Receptor Antagonist: A Phase 1 Randomized, Placebo‐Controlled Single‐ and Multiple‐Dose Escalation Study in Healthy Participants
Source: Clin Pharmacol Drug Dev. 2021 Jan 18;10(8):927–39. doi: 10.1002/cpdd.906 (PMC8451790; doi:10.1002/cpdd.906)
Supplement: Supplementary file 1 — Supporting Information. [file CPDD-10-927-s001.docx]

Supplementary Material for the manuscript titled:

Safety, Pharmacokinetics, and Pharmacodynamics of Trazpiroben (TAK-906), a Novel Selective D_2_/D_3_ Receptor Antagonist: A Phase 1 Randomized Placebo-Controlled Single and Multiple Dose-Escalation Study in Healthy Participants

Roger L. Whiting^1^, Borje Darpo^2^, Chunlin Chen^3^, Margaret Fletcher^4^, Dan Combs^5^, Hongqi Xue^2^, and Randall R. Stoltz^6^

## Statistical Methods for PK and PD Analyses

Data analyses were performed using statistical software SAS^®^ Version 9.3, WinNonlin Version 6.1 and Microsoft Excel 2010. All pharmacokinetic (PK) and pharmacodynamic (PD) parameters were summarized by cohort and study day using count, arithmetic mean, standard deviation (SD), coefficient of variance (% CV), minimum, maximum, median, and geometric mean and compared with pooled data from the placebo cohorts.

The PK analysis set included all participants who received at least one dose of study drug and for whom at least one post-dose sample result for trazpiroben was reported. The PD analysis set included all participants who received at least one dose of study drug and for whom at least one post-dose serum prolactin result was reported. Baseline for the PD analysis was the predose concentration on day 1 (single ascending dose [SAD] and multiple ascending dose [MAD] studies) and day 5 (MAD study only).

Analysis of dose-response for PK and PD parameters was conducted using three different methods: Method 1: dose proportionality of PK exposure parameters (C_max_ and AUC parameters) across all doses was determined using dose-adjusted C_max_ and AUC, calculated for each participant by dividing AUC and C_max_ by dose; analysis of variance (ANOVA) appropriate for a parallel, dose-ascending design was performed on the dose-adjusted parameters to assess dose proportionality, with dose as the main effect variable. Method 2: plots of C_max_ and AUC parameters versus dose were prepared with linear and power-curve regression lines superimposed on individual participant data and examined. Method 3: exposure ratios (ERs) of C_max_ and AUC parameters at adjacent doses as well as at the maximum and minimum doses administered, were compared to dose ratios (DRs). An ER/DR ratio close to 1.0 indicated dose proportionality. Deviations of less than 20% were considered minor.

Analysis of trazpiroben accumulation (in MAD study) was conducted by computing the ratios of trazpiroben C_max_, C_min_, C_avg_, and AUC for day 5 to day 1. Observed accumulation was compared to that predicted from theory (accumulation index). The approach to presumed steady-state was assessed from the predose and trough concentrations using graphical methods.

Evaluation of the time dependence of trazpiroben kinetics in the MAD study was conducted by comparing CL/F and *t*_1/2_ (non-exposure parameters) across study days in a manner similar to that for accumulation. Plots of non-exposure parameters versus dose were made to evaluate any changes in these parameters across the dose range studied. These parameters were also evaluated using ANOVA with dose as the main effect variable.

The effect of food on the PK of trazpiroben was determined with participants in the fasted versus fed state, in the SAD study 25 mg cohort, by comparison of the PK exposure parameters C_max_, AUC_0–12_, AUC_last_, and AUC_0–8_. The 90% confidence intervals (CIs) were computed on the ratio of the geometric least-squares means of log-transformed exposure parameters for fed (test) versus fasted (reference) treatments. An increase or decrease of less than 20% in the parameter was determined if the 90% CI contained the standard interval of 80–125%. If the 90% CIs were not entirely contained in this interval, then a food effect on PK exposure parameters could not be ruled out.

# *Bioanalytical Methods*

### *Plasma Trazpiroben Measurements*

Blood samples intended for trazpiroben concentration determination with K_2_EDTA anticoagulant were processed to plasma, stored at –70°C, and shipped frozen to the bioanalytical laboratory (Covance NWT West Trenton New Jersey). Samples were analyzed by one of two methods: high range or low range LC-MS/MS.

### Analyst software (version 1.4.2 and 1.6.1) was used for acquisition of LC-MS/MS data and integration of trazpiroben and warfarin or maraviroc-d^6^ chromatographic peaks. Watson LIMS (version 7.4.2) was used for laboratory information management including study design, sample receipt, interfacing with instruments, regressing analytical results, and data reporting. *High Range LC-MS/MS Method*

Blood was processed to plasma via protein precipitation as follows: 500 µL 1% formic acid in acetonitrile (ACN) was added to the sample which was then vortexed for 2–3 minutes followed by centrifugation at 3000 rpm for 5 min. A 300 µL aliquot of the supernatant was evaporated to dryness followed by reconstitution in 600 µL ACN:H_2_O:formic acid (50:50:0.1), and a 50 µL aliquot was injected into the LC-MS/MS.

Plasma samples were analyzed using a validated LC-MS/MS positive electro-spray method using warfarin as the internal standard. Runs 1 and 2 only used this method which had a quantitation range of 1.00–1000 ng/mL. All samples analyzed with the high range method that were below the lower limit of quantification (LLOQ) were re-analyzed using the low range method.

The high range calibration standard concentrations were 1.00, 2.00, 5.00, 10.0, 50.0, 200, 500, 900, and 1000 ng/mL. QC sample concentrations were 3.0, 30.0, 400.0, and 800.0 ng/mL. The method precision (%CV) was 2.4% to 14.6% and the accuracy (%Bias) was –5.3% to –1.3%.

Mass spectrometer instrument settings were as follows: trazpiroben Q1 mass 518.4, Q3 mass 244.2; warfarin internal standard Q1 mass 309.3, Q3 mass 163.2; HPLC column Fortis Diphenyl, 5 µm, 50 × 2.1 mm (Fortis); mobile phase: A 0.1% formic acid in water; B 0.1% formic acid in ACN; C tetrahydrofuran (THF). Ternary gradient initial flow was 0.300 mL/min of 65% B, 0% C.

### *Low Range LC-MS/MS Method*

Blood was processed to plasma via liquid-liquid extraction (low range method) as follows: 100 µL citrate buffer (pH 5), 800 µL methyl tert-butyl ether:ethyl acetate (50:50) was added to the sample, which was then mixed 13 times followed by centrifugation at 3000 rpm for 5 min. A 300 µL aliquot of the supernatant was evaporated to dryness and then reconstituted in 300 µL ACN:H_2_O:formic acid (40:60:0.l), and a 100 µL aliquot was injected into the LC-MS/MS.

Plasma samples were analyzed using a validated LC-MS/MS positive electro-spray method using maraviroc-d^6^ as the internal standard. Runs 3 through 30 used this method which had a quantitation range of 0.05–50.0 ng/mL.

The low range calibration standard concentrations were 0.0500, 0.100, 0.200, 0.500, 2.00, 5.00, 25.00, 45.00, and 50.00 ng/mL. QC sample concentrations were 0.15, 1.50, 20.00, and 40.00 ng/mL. The dilution factor was 10. The method precision (%CV) was 6.5% to 6.9% and the accuracy (%Bias) was –3.0% to –1.3%.

Mass spectrometer instrument settings were as follows: trazpiroben Q1 mass 518.4, Q3 mass 232.2; maraviroc internal standard Q1 mass 520.2, Q3 mass 389.2; HPLC column Gemini C6-Phenyl, 5 µm, 50 × 2.0 mm (Phenomenex); mobile phase: A methanol:H_2_O:1 M ABC (aq) (10:90:0.2); B ACN:H_2_O:1 M ABC (aq):NH_4_OH (90:10:0.2:0.1); C THF. Ternary gradient initial flow was 0.400 mL/min of 30% B, 0% C.

### *Serum Prolactin Measurements*

Blood samples intended for prolactin concentration determination were processed to serum, frozen and stored at –70°C until analysis. Serum samples were analyzed using the ADVIA Centaur^®^ analyzer (Siemens Healthcare Diagnostics Inc., Tarrytown NY) with a prolactin commercial assay kit for a direct chemiluminescent immunoassay. The assay is a two-site sandwich immunoassay using direct chemiluminometric technology, which uses constant amounts of two antibodies. The first antibody, in the Lite Reagent, is a polyclonal goat anti-prolactin antibody labeled with acridinium ester. The second antibody, in the Solid Phase, is a monoclonal mouse anti-prolactin antibody, which is covalently coupled to paramagnetic particles. The addition of hydroxyl groups complete the flash reaction by addition of acid and base solutions. The analyzer measures the chemical light reaction.

The assay range is 0.3–200 ng/mL. Assay precision (%CV) is less than 10%. Good accuracy has been determined for the kit by comparison to a separate assay (Bayer automated chemiluminescent immunoassay system Prolactin assay). Precision according to the manufacturer was tested in 6 samples spiked with 3.3–118 ng/mL prolactin in 6 replicates, each run 24 times. The within-run %CV was < 4.4%, the run-to-run %CV was < 5.3%, and the total %CV was < 6.6%.

The assay was tested for cross-reactivity to human somatotropic hormone, leutinizing hormone, human chorionic gonadotrophin, follicle-stimulating hormone, human growth hormone, and human placental lactogen, and prolactin concentrations with cross-reactants was nearly the same as without.

The ADVIA Centaur prolactin assay is correlated to the World Health Organization standard as ADVIA Centaur prolactin = 1.06 (WHO) – 0.6 IU/mL with a correlation coefficient of
r = 0.99. The assay conversion is 1.0 ng/mL = 21.2 µIU/mL.^1–3^

Procedure and reagents: to a 25 µL aliquot of serum sample, 100 µL Lite Reagent was added and incubated for 6 minutes at 37°C. Then 450 µL of Solid Phase was added and incubated for 2.5 minutes at 37°C. The mixture was then separated and aspirated into a wash cuvette with water. 300 µL each of Acid and Base reagent were added to initiate the chemiluminescent reaction. The amount of prolactin is proportional to the relative light units with quantitation against calibration standards.

Lite Reagent: 5.0 mL Ready Pack containing polyclonal goat anti-prolactin antibody (~0.16 µg/mL) labeled with acridinium ester in buffer with sodium azide (0.11%) and preservatives.

Solid Phase: 22.5 mL Ready Pack containing monoclonal mouse anti-prolactin antibody (~3.67 µg/mL) covalently coupled to paramagnetic particles in buffer with protein stabilizer, sodium azide (0.11%), and preservatives.

Multi-Diluent: 25.0 mL Ready Pack containing equine serum with sodium azide (0.1%) and preservatives.

Acid Reagent: 0.5% H_2_O_2_, 0.1 N HNO_3_.

Base Reagent: 0.25 N NaOH and surfactant.

## Nonclinical Evaluation of Trazpiroben

### *Pharmacology*

The pharmacology of trazpiroben has been well characterized in *in vitro* and *in vivo* nonclinical models in rats and dogs using the oral route of administration. *In vitro* studies of receptor binding affinity and activity, and *in vivo* studies of effects on prolactin secretion in rats and apomorphine‑induced emesis in dogs, have demonstrated primary pharmacologic actions of trazpiroben consistent with D_2_/D_3_ antagonism, with target engagement at oral doses of 0.1–1.0 mg/kg.

### *Nonclinical Pharmacokinetics and Metabolism*

The pharmacokinetics (PK) and metabolism of trazpiroben have been evaluated during *in vitro* and *in vivo* studies. Trazpiroben PK was dose proportional in rats and dogs and no accumulation was observed with repeat dosing. The absolute bioavailability of trazpiroben was approximately 19% in rats following oral gavage administration of trazpiroben, and up to 18% in dogs following oral capsule administration of trazpiroben. Trazpiroben is a substrate of p-glycoprotein and demonstrates low passive permeability. Brain penetration was determined to be minimal,

as demonstrated by the plasma to cerebrospinal fluid total trazpiroben concentration ratios of 700:1 in both rats and dogs at 1 hour post-dose, and of 260:1 in dogs at 3 hours post dose.

The metabolism of trazpiroben was evaluated *in vitro* using rat, dog, and human hepatocytes and microsomes, with no unique or disproportionate human metabolites observed.

Trazpiroben is unlikely to perpetrate drug-drug interactions (manuscript in preparation).

Toxicokinetic evaluations were performed as part of the pivotal GLP-compliant repeat dose toxicology studies in rats and dogs. In both rats and dogs, exposure (AUC) after single oral gavage doses increased in a greater than proportional manner when evaluated across the entire range of doses studied (3–1000 mg/kg in rats and 1–500 mg/kg in dogs). Exposure in female animals was generally greater than that in males but usually by less than two-fold.

### *Nonclinical Safety, Including Safety Pharmacology*

The safety of trazpiroben was tested in range-finding oral toxicity and pivotal 28-day repeated-dose toxicity studies in rats and dogs, and in a battery of genotoxicity assays, including an Ames assay and an *in vitro* chromosome aberration assay. Trazpiroben produced no relevant changes in cardiovascular parameter evaluations (human ether-a-gogo-related gene [hERG] and dog telemetry studies) or on the respiratory and central nervous systems. Trazpiroben did not show genotoxicity in the standard battery of tests.

In Sprague-Dawley rats, trazpiroben doses of 0, 100, 300, or 1000 mg/kg/day were administered once daily for 28 days via oral gavage, followed by a 2-week treatment-free recovery period. No remarkable clinical observations, ophthalmic findings, or effects on body weight, food consumption, or clinical pathology were noted. Feminization of the mammary gland in males, and hypertrophy of the mammary gland and mucification of the vaginal and cervical epithelium in females, were noted at doses of 100, 300, and 1000 mg/kg/day. These effects were not considered dose-limiting at any dose level; they were considered to be secondary to prolactin elevations associated with the pharmacological action of trazpiroben. The no-observable-adverse-effect level was considered to be 1000 mg/kg/day, corresponding to C_max_ values of 16 700–23 500 ng/mL and AUC_0-6_ values of 69 500–122 000 ng•h/mL for males and females, respectively, following 28 daily oral gavage doses of trazpiroben in Sprague-Dawley rats.

In sexually immature beagle dogs, trazpiroben doses of 0, 1, 10, or 50 mg/kg/day were administered once daily for 28 days via oral capsule, followed by a 2-week dose-free recovery period. Trazpiroben-related clinical observations included intermittent or continuous tremors, ataxia, and eye abnormalities. These clinical observations were seen predominantly at the high dose (50 mg/kg/day), were observed at a lower incidence and lower severity at 10 mg/kg/day, and were virtually absent at 1 mg/kg/day. At 1 mg/kg/day, a single occurrence of mild tremors was observed in one female on Day 1. In dog, 1 mg/kg/day is considered a lowest-observable-effect dose (LOEL) due to this finding. Clinical observations noted in this study, which were considered to be consistent with mild sedation, were not noted at the end of the 2-week recovery period. Trazpiroben-related microscopic findings consisted of minimal to slight uterine maturation delay in females administered 1, 10, or 50 mg/kg/day: a global decrease in uterine cross-sectional diameter, fewer glands, and compact stroma and muscular tissue were observed relative to the control animals. This finding correlated with decreases in mean absolute and relative uterus weights in females administered ≥10 mg/kg/day, and with a macroscopic finding of small uterus in one female administered 50 mg/kg/day. Females in all dose groups were considered to be of immature reproductive status, as evidenced by a lack of ovarian corpora lutea and a lack of cyclic variation of the vaginal epithelium. Slight uterine maturation delay following the 2-week dose-free recovery period was observed in one female who had been administered 50 mg/kg/day. Both the clinical observations and the delay in uterine maturation were consistent with the expected pharmacologic activity of this pharmaceutical class and were not considered dose-limiting under the conditions of this study. The no-observed-adverse-effect-level of trazpiroben was considered to be 50 mg/kg/day, corresponding to mean C_max_ values of 4340 and 1900 ng/mL and AUC_0-t_ values of 10800 and 5880 ng•h/mL for males and females, respectively, following 28 daily oral capsule doses of trazpiroben in beagle dogs.

Trazpiroben produced consistent increases in serum concentrations of prolactin in both males and females and in both rats and dogs. These studies indicated that the microscopic findings noted in the 28-day repeat-dose toxicity studies in rats and dogs were secondary to hyperprolactinemia and consistent with the known pharmacological activity of trazpiroben.

Given the low blood–brain barrier penetrability of trazpiroben and the high doses of trazpiroben that were administered in the toxicology studies, the proposed human starting dose of 5 mg in a 60‑kg person (0.08 mg/kg) is not expected to result in sufficient central nervous system concentrations of trazpiroben to produce adverse effects. Prolactin-mediated effects, as well as potential mild sedation, are readily monitored in clinical studies and are reversible.

## References

1. Hendriks HA, Kortlandt W, Verweij WM. Analytical performance comparison of five new generation immunoassay analyzers. *Ned Tijdschr Klin Chem* 2000;25(3):170–7.
2. Beltran L, Fahie-Wilson MN, McKenna TJ, Kavanagh L, Smith TP. Serum total prolactin and monomeric prolactin reference intervals determined by precipitation with polyethylene glycol: evaluation and validation on common immunoassay platforms. *Clinical Chemistry* 2008;54(10):1673–81.
3. Schüring AN, Kelsch R, Pierściński G, Nofer J-R. Establishing reference intervals for sex hormones on the analytical platforms Advia Centaur and Immulite 2000XP. *Ann Lab Med* 2016;36:55–9.

Supplementary Table 1. Placebo-corrected change from baseline in heart rate and QTcF in the single ascending dose study.

| **Parameter** | **Nominal time after dosing (h)** | **Statistic** | **Trazpiroben dose (fed/fasted state)** | | | | | | | |
| --- | --- | --- | --- | --- | --- | --- | --- | --- | --- | --- |
|  |  |  | **5 mg (fasted)** | **10 mg (fasted)** | **25 mg (fasted; day 1)** | **25 mg fed (day 3)** | **50 mg (fasted)** | **100 mg (fasted)** | **200 mg (fasted)** | **300 mg (fasted)** |
| △△HR, bpm | 0.25 | LS mean | –1.7 | –1.7 | –0.6 | 8.2 | –2.7 | –1.0 | –3.3 | 1.2 |
|  |  | SE | 2.30 | 2.19 | 2.30 | 2.19 | 2.19 | 2.19 | 2.34 | 2.19 |
|  |  | 90% CI | –5.5, 2 | –5.3, 2 | –4.4, 3.2 | 4.6, 11.8 | –6.3, 1 | –4.6, 2.6 | –7.2, 0.5 | –2.4, 4.8 |
|  | 0.5 | LS mean | –2.2 | –2.6 | –4.4 | 5.4 | –3.7 | –2.2 | –1.2 | –1.1 |
|  |  | SE | 2.16 | 2.16 | 2.16 | 2.16 | 2.16 | 2.16 | 2.31 | 2.16 |
|  |  | 90% CI | –5.8, 1.3 | –6.1, 1 | –8, –0.9 | 1.9, 9 | –7.3, –0.2 | –5.8, 1.3 | –5, 2.6 | –4.6, 2.5 |
|  | 1 | LS mean | 0.3 | –0.0 | –5.0 | 4.5 | –3.4 | –1.1 | 1.0 | 0.3 |
|  |  | SE | 2.17 | 2.17 | 2.17 | 2.17 | 2.17 | 2.29 | 2.32 | 2.17 |
|  |  | 90% CI | –3.3, 3.9 | –3.6, 3.5 | –8.6, –1.5 | 0.9, 8 | –6.9, 0.2 | –4.9, 2.6 | –2.9, 4.8 | –3.3, 3.9 |
|  | 1.5 | LS mean | –1.5 | –1.5 | –1.7 | 6.5 | –1.9 | –1.0 | 1.2 | 1.6 |
|  |  | SE | 2.16 | 2.16 | 2.16 | 2.16 | 2.16 | 2.16 | 2.31 | 2.16 |
|  |  | 90% CI | –5.1, 2 | –5.1, 2 | –5.2, 1.9 | 2.9, 10 | –5.4, 1.7 | –4.6, 2.5 | –2.6, 5 | –1.9, 5.2 |
|  | 2 | LS mean | –0.7 | –3.7 | –0.6 | 7.3 | –3.4 | –1.1 | 0.4 | 2.6 |
|  |  | SE | 2.16 | 2.16 | 2.16 | 2.16 | 2.16 | 2.27 | 2.31 | 2.16 |
|  |  | 90% CI | –4.3, 2.8 | –7.3, –0.2 | –4.1, 3 | 3.7, 10.8 | –7, 0.1 | –4.8, 2.7 | –3.4, 4.2 | –1, 6.1 |
|  | 3 | LS mean | –0.8 | –1.7 | –2.2 | 5.7 | –4.5 | 0.7 | –2.3 | 2.3 |
|  |  | SE | 2.16 | 2.16 | 2.16 | 2.16 | 2.16 | 2.16 | 2.31 | 2.16 |
|  |  | 90% CI | –4.4, 2.7 | –5.2, 1.9 | –5.7, 1.4 | 2.1, 9.2 | –8.1, –0.9 | –2.9, 4.2 | –6.1, 1.5 | –1.2, 5.9 |
|  | 4 | LS mean | 0.2 | –0.9 | –1.1 | 5.9 | –3.4 | –1.3 | –1.2 | 2.7 |
|  |  | SE | 2.16 | 2.16 | 2.27 | 2.16 | 2.16 | 2.16 | 2.31 | 2.27 |
|  |  | 90% CI | –3.3, 3.8 | –4.5, 2.6 | –4.8, 2.7 | 2.3, 9.5 | –7, 0.1 | –4.8, 2.3 | –5, 2.6 | –1.1, 6.4 |
|  | 6 | LS mean | 0.5 | 0.8 | –5.5 | –1.5 | –4.0 | 0.9 | –2.4 | –0.7 |
|  |  | SE | 2.17 | 2.17 | 2.17 | 2.17 | 2.17 | 2.29 | 2.32 | 2.17 |
|  |  | 90% CI | –3.1, 4.1 | –2.8, 4.4 | –9.1, –1.9 | –5.1, 2.1 | –7.6, –0.4 | –2.8, 4.7 | –6.2, 1.5 | –4.3, 2.9 |
|  | 8 | LS mean | 2.0 | 2.5 | 0.3 | –0.0 | –3.2 | –0.2 | –0.7 | –1.2 |
|  |  | SE | 2.16 | 2.16 | 2.16 | 2.16 | 2.16 | 2.16 | 2.31 | 2.27 |
|  |  | 90% CI | –1.6, 5.5 | –1.1, 6 | –3.3, 3.9 | –3.6, 3.5 | –6.8, 0.4 | –3.8.3.4 | –4.5, 3.1 | –5, 2.5 |
|  | 12 | LS mean | –0.5 | 0.8 | –5.5 | –2.5 | –5.0 | –3.3 | –1.4 | –2.7 |
|  |  | SE | 2.17 | 2.17 | 2.17 | 2.17 | 2.17 | 2.29 | 2.32 | 2.17 |
|  |  | 90% CI | –4.1, 3.1 | –2.7, 4.4 | –9.1, –1.9 | –6.1, 1.1 | –8.6, –1.4 | –7, 0.5 | –5.2, 2.5 | –6.2, 0.9 |
|  | 24 | LS mean | –1.0 | –2.5 | –1.2 | 1.3 | –3.5 | 3.0 | –0.6 | –0.5 |
|  |  | SE | 2.16 | 2.16 | 2.16 | 2.16 | 2.16 | 2.16 | 2.31 | 2.16 |
|  |  | 90% CI | –4.6, 2.5 | –6.1, 1 | –4.7, 2.4 | –2.2, 4.9 | –7.1, 0 | –0.6, 6.5 | –4.4, 3.2 | –4.1, 3 |
| △△QTcF, ms | 0.25 | LS mean | 3.2 | –1.1 | 1.1 | –4.3 | 1.1 | 0.6 | –1.0 | –1.0 |
|  |  | SE | 3.0 | 2.9 | 3.0 | 2.9 | 2.9 | 2.9 | 3.0 | 2.9 |
|  |  | 90% CI | –1.8, 8.2 | –5.8, 3.6 | –3.8, 6.1 | –9, 0.4 | –3.6, 5.8 | –4.1, 5.3 | –6, 4 | –4.1, 5.3 |
|  | 0.5 | LS mean | 1.2 | 0.5 | –1.3 | –0.5 | 0.2 | 0.9 | 3.8 | 0.9 |
|  |  | SE | 2.8 | 2.8 | 2.8 | 2.8 | 2.8 | 2.8 | 3.0 | 2.8 |
|  |  | 90% CI | –3.4, 5.8 | –4.1, 5.2 | –5.9, 3.3 | –5.1, 4.2 | –4.4, 4.8 | –3.7, 5.5 | –1.1, 8.8 | –3.7, 5.5 |
|  | 1 | LS mean | 5.8 | 2.8 | 4.7 | 0.7 | 3.7 | 3.1 | 8.8 | 4.5 |
|  |  | SE | 2.8 | 2.8 | 2.8 | 2.8 | 2.8 | 3.0 | 3.0 | 2.8 |
|  |  | 90% CI | 1.2, 10.5 | –1.8, 7.5 | 0, 9.3 | –4, 5.3 | –1, 8.3 | –1.8, 8 | 3.8, 13.8 | –0.2, 9.2 |
|  | 1.5 | LS mean | 4.9 | 2.2 | 3.2 | –1.3 | 2.5 | 1.0 | 8.3 | 6.2 |
|  |  | SE | 2.8 | 2.8 | 2.8 | 2.8 | 2.8 | 2.8 | 3.0 | 2.8 |
|  |  | 90% CI | 0.2, 9.5 | –2.4, 6.8 | –1.4, 7.8 | –5.9, 3.3 | –2.1, 7.2 | –3.6, 5.7 | 3.3, 13.2 | 1.6, 10.8 |
|  | 2 | LS mean | 4.4 | 0.7 | 2.7 | –3.1 | 3.9 | 3.1 | 9.6 | 5.6 |
|  |  | SE | 2.8 | 2.8 | 2.8 | 2.8 | 2.8 | 3.0 | 3.0 | 2.8 |
|  |  | 90% CI | –0.2, 9 | –3.9, 5.4 | –1.9, 7.4 | –7.7, 1.5 | –0.7, 8.5 | –1.8, 8 | 4.7, 14.6 | 0.9, 10.2 |
|  | 3 | LS mean | 2.2 | 1.3 | 0.8 | –5.2 | 4.3 | 2.2 | 3.8 | 2.2 |
|  |  | SE | 2.8 | 2.8 | 2.8 | 2.8 | 2.8 | 2.8 | 3.0 | 2.8 |
|  |  | 90% CI | –2.5, 6.8 | –3.3, 5.9 | –3.8, 5.4 | –9.8, –0.6 | –0.3, 8.9 | –2.5, 6.8 | –1.2, 8.7 | –2.5, 6.8 |
|  | 4 | LS mean | 2.9 | 2.2 | 4.0 | 0.4 | 6.4 | 0.2 | 6.7 | 4.3 |
|  |  | SE | 2.8 | 2.8 | 3.0 | 2.8 | 2.8 | 2.8 | 3.0 | 3.0 |
|  |  | 90% CI | –1.8, 7.5 | –2.4, 6.8 | –0.9, 8.8 | –4.3, 5 | 1.7, 11 | –4.4, 4.8 | 1.7, 11.6 | –0.6, 9.2 |
|  | 6 | LS mean | –3.0 | –6.8 | –8.5 | –2.5 | –5.0 | –0.9 | 0.4 | –4.5 |
|  |  | SE | 2.8 | 2.8 | 2.8 | 2.8 | 2.8 | 3.0 | 3.0 | 2.8 |
|  |  | 90% CI | –7.7, 1.7 | –11.5, –2.2 | –13.2, –3.8 | –7.2, 2.2 | –9.7, –0.3 | –5.8, 4 | –4.6, 5.4 | –9.2, 0.2 |
|  | 8 | LS mean | 2.7 | –0.4 | –1.3 | 2.2 | 2.9 | –0.6 | 4.3 | –3.7 |
|  |  | SE | 2.8 | 2.8 | 2.8 | 2.8 | 2.8 | 2.8 | 3.0 | 3.0 |
|  |  | 90% CI | –1.9, 7.4 | –5.1, 4.2 | –5.9, 3.4 | –2.4, 6.9 | –1.7, 7.5 | –5.2, 4 | –0.6, 9.3 | –8.6, 1.2 |
|  | 12 | LS mean | –2.3 | –5.3 | –6.0 | 3.4 | 1.7 | –6.0 | 3.1 | –3.5 |
|  |  | SE | 2.8 | 2.8 | 2.8 | 2.8 | 2.8 | 3.0 | 3.0 | 2.8 |
|  |  | 90% CI | –7, 2.4 | –10, –0.6 | –10.6, –1.3 | –1.3, 8 | –3, 6.4 | –10.9, –1 | –1.8, 8.1 | –8.1, 1.2 |
|  | 24 | LS mean | 4.0 | 1.5 | 1.0 | 7.9 | 4.0 | 0.5 | 6.2 | 0.5 |
|  |  | SE | 2.8 | 2.8 | 2.8 | 2.8 | 2.8 | 2.8 | 3.0 | 2.8 |
|  |  | 90% CI | –0.6, 8.7 | –3.1, 6.2 | –3.6, 5.7 | 3.2, 12.5 | –0.6, 8.7 | –4.1, 5.2 | 1.2, 11.1 | –4.1, 5.2 |

CI, confidence interval; HR, heart rate; LS, least-squares; △△QTcF, placebo-corrected change in QT interval corrected for heart rate using Fridericia’s correction; SE, standard error.
